# Tigecycline application in a 3-month-old infant with multiple drug resistant *Klebsiella pneumonia*: a case report

**DOI:** 10.1186/s13099-018-0253-x

**Published:** 2018-06-22

**Authors:** Cheng Peng, Xiaofeng Wang, Jiangwei Zhang, Yi Jiang, Xinlin Hou

**Affiliations:** Department of Neonatal Ward, Peking University First Hospital, No. 1, Xi Anmen Street, Xicheng District, Beijing, 100034 China

**Keywords:** Multiple drug resistant, *Klebsiella pneumoniae*, Tigecycline, Pediatric

## Abstract

**Background:**

Tigecycline is an ‘immature’ antibiotic for children. We report the youngest surviving patient who received a complete tigecycline treatment, and no significant adverse effects occurred in the patient.

**Case presentation:**

The 3-month old infant suffered from a catheter-associated bloodstream infection by multiple drug resistant *Klebsiella pneumoniae*. Tigecycline was considered as a salvage therapy to control the severe sepsis. The therapy consisted of 3 mg/kg as a loading dose and 1.5 mg/kg Q12 h as a maintenance dose for 26 days.

**Conclusion:**

Current researches are limited in clinical trials directly focused on children. This therapeutic schedule might be safe for patients who are above 3 months old.

**Electronic supplementary material:**

The online version of this article (10.1186/s13099-018-0253-x) contains supplementary material, which is available to authorized users.

## Background

*Klebsiella pneumoniae* belongs to the family *Enterobacteriaceae*. The microbe is generally found as colonized bacteria, but is a latent agent for fatal infections. It is one of the most clinically relevant species in nosocomial infections and immunosuppressed individuals, especially in neonates and premature infants. In 2013, it was the second leading cause of Gram-negative bloodstream infection in Europe, after *Escherichia coli* [1]. The 3-month-old patient who suffered from a catheter-associated bloodstream infection by multiple drug resistant *Klebsiella pneumoniae*. Tigecycline is an ‘immature’ antibiotic for children. Pediatricians must give careful consideration to several aspects of using this antibiotic, for instance, the dosage, the course, the side effects, etc. Existing literature review concluded the dosage should be 1 mg/kg every 12 h in children above age 12 years [2], but the dosage was limited for infants on that occasion. We consulted the dosage for adult with initial 150 mg IV, followed by 75 mg IV every 12 h [3]. After the conversion of a 50 kg bodyweight, we empirically decided on 3 mg/kg as a loading dose and 1.5 mg/kg Q12 h as a maintenance dose, for 26 days. We report the youngest surviving patient who received a complete tigecycline treatment, and no significant adverse effects occurred in the patient.

## Case presentation

After the detection of a suspicious hydramnios and the premature rupture of the amniotic membranes, a male infant was born by caesarean section at 38 weeks with a weight of 2900 g. He was the smaller of a set of identical twins.

The patient was being breastfed shortly after his birth and began vomiting intermittently after 12 h. At the age of 6 days, he was clinically suspected to be afflicted by small intestinal atresia and Hirschsprung’s disease and underwent an enterectomy and an ileostomy. His identical twin brother was diagnosed with the same disease and received a similar operation. The patient was under fasting for about 1 week after surgery. Afterwards, he gradually began breast feeding, from 5–15 ml to 25 ml each time, every 2–3 h while continuing part of the intravenous nutrition program (about 200–300 ml per day using central venous catheter). He had intermittent vomiting of yellow-green liquid and the stoma continuously discharged thin paste-like yellow-green objects, ranging in quantity from 70–240 ml per day. He also suffered from sepsis to was given vancomycin, meropenem and caspofungin for anti-infective therapy. Despite this treatment, the patient continued vomiting occasionally and his weight could not grow as expected in the following 3 months, hence he was transferred to our hospital for further treatment.

On admission, his body weight was 3000 g and his height was 50 cm (Weight for height Z score was − 1). Physical examination revealed severe malnutrition and the absence of most subcutaneous fat. Abdominal distention was obvious and bilateral separated stomas could be seen above the navel. His body temperature was 37.5 °C, his pulse was 146 beats/min, his respiratory rate was 38 breaths/min, and his blood pressure was 75/40 mmHg. The white blood cell (WBC) count was 11.83 × 10^9^/l and C-reactive protein (CRP) level was 88 mg/l. The infection could be controlled by intravenous nutrition and an application of vancomycin and meropenem for anti-infection.

**Fig. 1:**
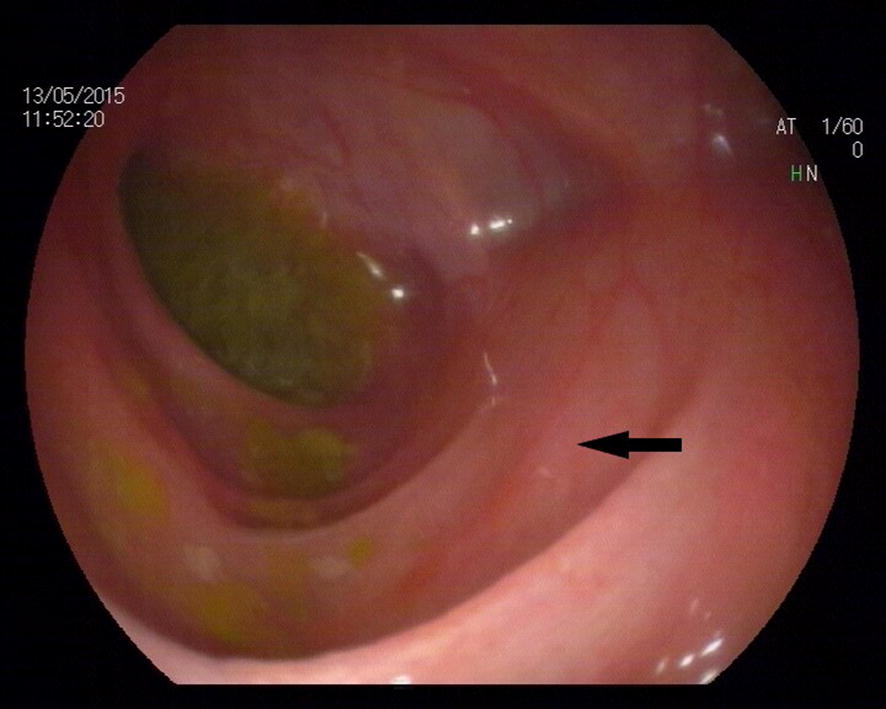
Colonoscopy graphic (transverse colon). The black arrow shows a smooth intestinal wall and clear vessels

**Fig. 2:**
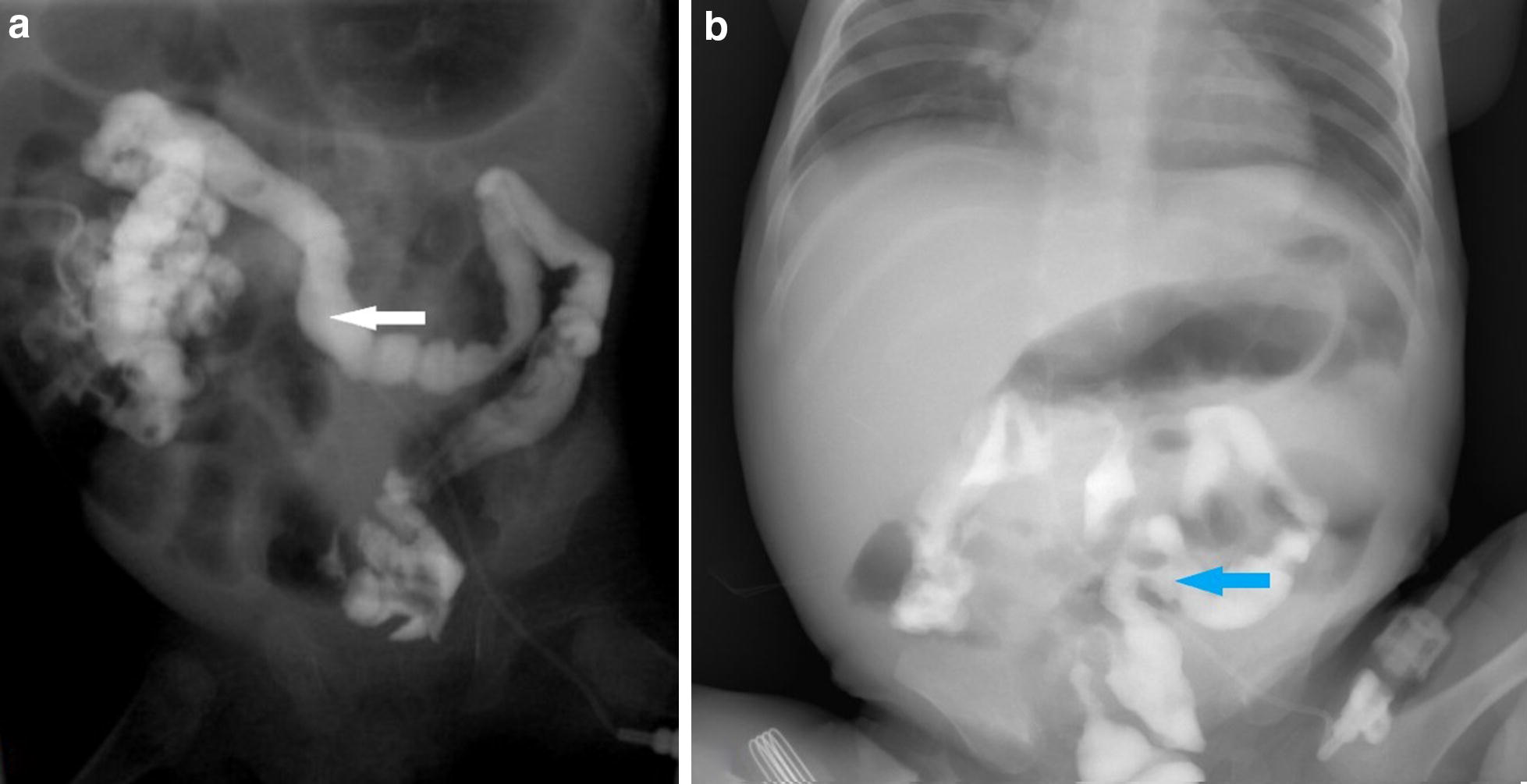
**a** The distal stoma imaging. The maximum width of the transverse colon was approximately 0.8 cm (the white arrow). **b** The upright plain abdominal radiograph. The image was taken 24 h after the previous imaging. The contrast medium was stranded in the colon (the blue arrow)

After the infant started receiving feeding, he suffered from a relapsing infection. WBC count and CRP level increased to 28.9 × 10^9^/l and 156 mg/l, respectively. The temperature, WBC, and CRP returned to an acceptable range as vancomycin and meropenem (20 mg/kg, Q8 h) were given. A colonoscopy was performed on the infant, which indicated the smooth intestinal mucosa and clear intestinal wall vessels (Fig. 1). A distal stoma imaging was also performed on the infant, which revealed a rigid lumen from the transverse colon to the descending colon (Fig. 2).

A 4th-time infection occurred with the expiration of the central venous catheter (CVC). The patient got a fever with chills. It was highly likely that the patient caught a catheter-associated bloodstream infection, and the corresponding catheter tip cultures revealed multiple drug resistant *Klebsiella pneumoniae*, which was sensitive to tigecycline, polymyxin E, and cotrimoxazole. The minimum inhibitory concentration (MIC) for tigecycline was ≤ 0.25. A laboratory test showed WBC count 2.73 × 10^9^/l, CRP 73 mg/l, Platelet count 31 × 10^9^/l and procalcitonin (PCT) 4.03 ng/ml. Based on the results, we changed to new antiseptic-impregnated central venous catheters with hibitane and sulfadiazine. At the same time, we administered tigecycline (3 mg/kg as a loading dose and 1.5 mg/kg, Q12 h as a maintenance dose, 26 days), cotrimoxazole (20 mg/kg, Q12 h, 35 days) and polymyxin E (20,000 IU/kg Q8 h, 5 days). To prevent cocci infection, linezolid and rifampicin (10 mg/kg, Q12 h) were used for a short period. The liver function, renal function and coagulation profile were taken periodically (see Additional file 1). The liver function was probably affected by long-term intravenous nutrition and vancomycin (Additional file 1: Table S1). The renal function was normal (Additional file 1: Table S2). The coagulation function showed a prolonged activated partial thromboplastin time (APTT) without specific value, which might result from the blood product support and the severe infection (Additional file 1: Table S3). The result did not point out a disseminated intravascular coagulation, so the patient went for the enterotomy to control the infection.

During the intensive anti-infective treatment, a blood culture was taken. The test was positive for the microbe *Candida parapsilosis*. We decided to withdraw caspofungin and add amphotericin B liposome (3 mg/kg, 21 days) –the previous antifungal drugs were fluconazole and caspofungin. Blood products and hepatic protectants were infused to resist septic shock and liver injury. The patient’s identical twin brother was also infected by multiple drug resistant *Klebsiella pneumoniae* sepsis and passed away on account of septic shock before blood culture results came back, the brother was not given tigecycline.

Considering the recurrent infections, the patient received intestinal anastomosis and ileostomy. The surgical findings revealed a 10 cm stenotic small intestine in the proximal stoma. The pediatric surgeon excised the repeatedly infected segment and retained about 100 cm of the residual small intestine. The pathological tissue helped us to diagnose intestinal neuronal dysplasia (IND).

**Fig. 3:**
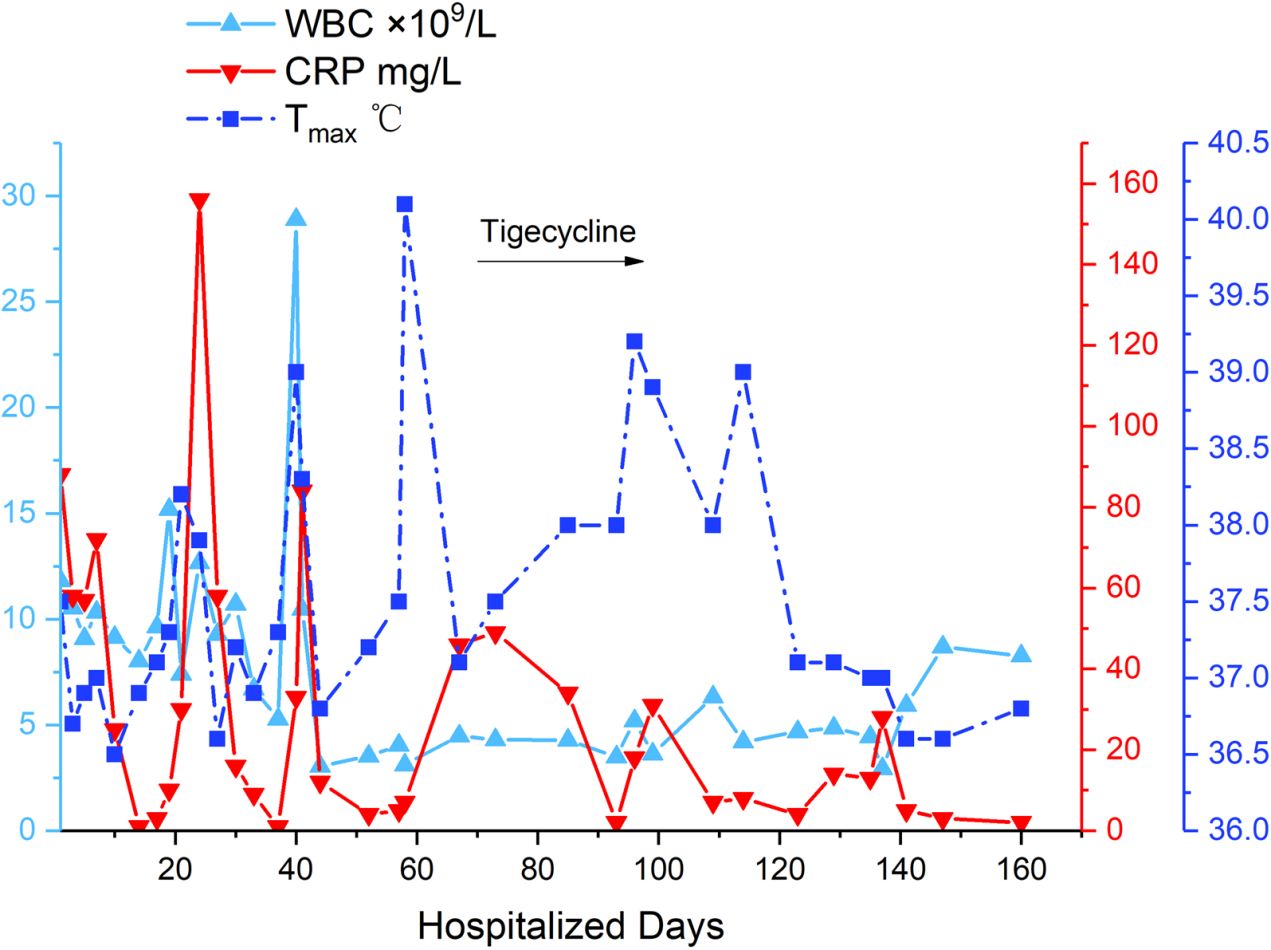
The clinical characteristics of the patient. The black arrow showed that tigecycline was applied from day 70 to day 95

We offered meticulous postoperative support and the CBC became normal (WBC count 6.86 × 10^9^/l, neutrophil% 57.9%, lymphocyte % 30.8%, hemoglobin 119 g/l, platelets 126 × 10^9^/l, CRP 4 mg/l). The pathological findings manifested intestinal neuronal dysplasia. Gradually, the patient recovered and started oral feeding 10 days after surgery. The patient’s temperature, CRP and WBC are shown in Fig. 3. In spite of abdominal distension and diarrhea at times, the patient’s body weight did not decrease. After combining the powdered milk with supplementary food and oral rehydration salts, defecation and emiction were satisfactory and serum electrolytes were normal. In the period of close observation, no significant early or late side effects occurred in the patient, such as GI symptoms, acute pancreatitis, or discoloration of the teeth.

Currently the patient is 34 months old, with a weight of 14 kg, and a height of 90 cm. He has 20 complete deciduous teeth, none of which are discolored. He suffered from an acute bacterial diarrhea when he was 18 months old, but recovered in 1 week after he was given meropenem. 32 months after birth, he suffered from rotavirus diarrhea and severe dehydration which was cured after receiving intravenous fluid therapy. A recent visit showed that the peripheral hemoglobin was 126 g/l. The patient drinks Neocate 500 ml/day, and some supplementary food for daily nutrition after discharge. He defecates 200–300 ml every day, using oral rehydration salts as a supplement. His urine volume is about 1.5–2 ml/kg h.

## Discussion and conclusions

The life-threatening bacteria found in the patient’s bloodstream was *Klebsiella pneumoniae*, which could tolerate cephalosporin, carbapenem, aminoglycosides and quinolones [4]. The mechanisms of the antimicrobial resistance can be divided into four dimensions. (1) The β-lactamase–*Klebsiella pneumoniae* could generate almost all kinds of lactamases in accordance with the extensive application of cephalosporin. (2) Carbapenemases–Carbapenem is a class of effective antibiotic for *Klebsiella pneumoniae*, but the carbapenemases could lead to the dissemination the multi-drug resistant strains [5]. (3) Biofilm–Biofilm is a protective pattern of clustered bacteria covered by a polysaccharide matrix which contains pipes for transporting nutrition. The binding site of the antibiotics on the cover of *Klebsiella pneumoniae* may decrease in the biofilm environment [6]. (4) Efflux pump–there could exist a series of energy-dependent efflux pumps in one strain [7]. The overexpression of those pumps has been recognized as an important mechanism in the drug-resistant system.

Antimicrobial resistance is becoming increasingly concerning in public health. Multiple drug resistant infections remain difficult to suppress, for limited efficacious antibiotics could be applied in clinical practices. Carbapenem, glycopeptide, oxazolidinone, and such series of antibiotics are commonly used for infants in those urgent circumstances. The potential danger of super bacteria is alarming, physicians may not use any effective medicine in their treatments, regardless of meropenem, vancomycin, or linezolid etc. The 15-days mortality of carbapenem-resistant *Klebsiella pneumoniae* could reach up to 26% [8]. It could be more fatal for neonates with a weaker immune system compared with elder children and adults.

In retrospect, the patient in this case had indicators of intractable multidrug resistant bacteria. (1) The defect of the gut immunity. (2) The anastomotic stoma stenosis. (3) The long-term indwelling of CVC. (4) The mixed use of varieties of antibiotics. The pathological process is explained below: The ileostomy and the postoperative stoma stenosis led to the dysfunction of the gastrointestinal motility, this, coupled with the hypoimmunity condition, exacerbated the problem of intestinal bacterial translocation. Although we gave precise nursing with the CVC, the catheter-associated blood-stream infection was still hard to avoid. The longer the catheter remained in use, the higher probability of infection occurring. As a result, different combinations of antibiotics were served, which aggravated the growth of multidrug resistant bacteria. This vicious circle ultimately caused the life-threatening situation of the patient and his brother’s death. The enteric bacilli infected both of the twins. It was not the simple colonization in specific location on the body, but a severe pathogenic course.

As the case presentation described above, the *Klebsiella pneumoniae* showed resistance to cephalosporins, carbapenems, aminoglycosides, and quinolones while it was only sensitive to tigecycline, polymyxin E, and cotrimoxazole. Using tigecycline was a huge challenge for our team, because we have no experience with the drug. Tigecycline is one of the members of the glycylcyclines. As the next-generation analogue, the mechanism of the agent is similar with the tetracycline—the therapeutic effect is reversibly binding to the 30S ribosomal subunit and forming a blockade of the protein translation [9]. As a result, the peptide synthesis of the bacteria is inhibited. In spite of its classic bacteriostasis, recent studies are likely to prove the bactericidal effect in vitro against Gram-positive and Gram-negative bacteria [10, 11]. The expanded broad-spectrum activity has brought it up as a salvage therapy for infections [12]. In this case, the critical sepsis of *Klebsiella pneumoniae* could not be inhibited without applying infrequent antibiotics to the infants. A terrible cost was the death of his brother, who was infected by the same bacteria.

Researchers have found that the most common side effects of tigecycline are digestive symptoms, especially nausea, vomiting, and diarrhea [13, 14]. In the case described above, the patient showed intermittent vomiting after the operation. However, it was the operation that made it difficult for us to distinguish the intestinal dysfunction from a side effect of the tigecycline. Although severe adverse effects do not happen frequently among child patients, clinicians should also be concerned about acute pancreatitis [15, 16]. The mechanism might be the toxic metabolites, hypertriglyceridemia, or a high biliary concentration. In this case, the patient was 3 months old and could not express his abdominal discomfort directly. Abdominal ultrasound was taken, nevertheless, the laboratory test on the serum amylase and lipase was unnecessary. Another long-term side effects should not be forgotten–teeth discoloration and delay in ossification [17]. It was an important consideration for the patient. The new antibiotic is semisynthetically derived from minocycline by substituting a 9-t-butylglycylamido group at the 9 position on the D ring, and former clinical studies have proved its danger, so we have to take that into account. If the teeth were influenced by tigecycline, the quality of life might be significantly decreased. During the long-term follow-up, we observed that there was no significant teeth discoloration. The child is 3 years old now and his height is now 92 cm. The latest follow-up visit showed that the patient grew complete deciduous teeth and no discoloration presented itself.

However, confined to several reasons, the application of tigecycline are prudent: (1) Indication: due to the lack of experience for infant dose, clinical use is beyond the scope of application. (2) Medical expenses: Chinese children’s medical expenses need to be paid by parents first, and may be reimbursed in the future, but it is uncertain. (3) Concerns about long-term prognosis: parents do not have a clear understanding of the side effects of this drug, which brings up a lot of concerns. (4) Confined to the laboratory facility, we have no method to examine the plasma drug concentration.

Tigecycline is a rescuer for severe infections, but is still an ‘immature’ antibiotic for children. Pediatricians face many difficulties in judging an appropriate dosage and course for satisfying the pharmacodynamics and the adverse effects simultaneously. Current researches are limited in clinical trials directly focused on children [18]. Tigecycline is generally combined with other antibiotics in previous cases, so formulating a correct therapy is difficult. Researchers need a relatively long time to assess the optimal dose for various age groups.

In summary, we are confined to our inexperience, but considering our endeavor, it might be safe for children to consume 3 mg/kg as a loading dose and 1.5 mg/kg Q12 h as a maintenance dose. This case was the first and only case which the child used Tigecycline and has been cured in our institution, followed up until now. Further applications are required to meet the augmented demands on tigecycline application. When similar patients that have the indication for tigecycline are hospitalized, the dosage can be imitated and we will observe carefully on the antibiotic effects and side effects.

## Additional file

**Additional file 1: Table S1.** Liver function. **Table S2.** Renal function. **Table S3.** Coagulation function.

## Abbreviations

WBC: white blood cell
CRP: C-reactive protein
CVC: central venous catheter
MIC: minimum inhibitory concentration
PCT: procalcitonin
IND: intestinal neuronal dysplasia
ESBLs: extended-spectrum beta-lactamases
ALT: alanine transaminase
AST: aspartate transaminase
ALP: alkaline phosphatase
GGT: gamma glutamyl transpeptidase
ALB: albumin
TBIL: total bilirubin
SCr: serum creatinine
BUN: blood urea nitrogen
