# Supplementary material for: Tigecycline application in a 3-month-old infant with multiple drug resistant Klebsiella pneumonia: a case report
Source: Gut Pathog. 2018 Jun 22;10:25. doi: 10.1186/s13099-018-0253-x (PMC6014034; doi:10.1186/s13099-018-0253-x)
Supplement: Supplementary file 1 — Additional file 1: Table S1. Liver function. Table S2. Renal function. Table S3. Coagulation function. [file 13099_2018_253_MOESM1_ESM.docx]

Table S1: Liver function.

| Date | ALT IU/L | AST IU/L | ALP IU/L | GGT IU/L | ALB g/L | TBIL μmol/L |
| --- | --- | --- | --- | --- | --- | --- |
| Day 4 | 47 | 41 | 357 | 63 | 31.0 | 15.5 |
| Day 10 | 77 | 41 | 444 | 73 | 28.5 | 17.5 |
| Day 16 | 142 | 98 | 397 | 99 | 26.7 | 17.7 |
| Day 24 | 96 | 66 | 414 | 147 | 35.5 | 13.0 |

The patient’s liver function was abnormal without an elevated bilirubin, which was probably affected by long-term intravenous nutrition and vancomycin application.

ALT: alanine transaminase; AST: aspartate transaminase; ALP: alkaline phosphatase; GGT: gamma glutamyl transpeptidase; ALB: albumin; TBIL: total bilirubin.

Table S2: Renal function.

| Date | SCr μmol/L | BUN mmol/L |
| --- | --- | --- |
| Day 4 | 23.00 | 4.52 |
| Day 10 | 30.60 | 6.64 |
| Day 16 | 20.90 | 4.37 |
| Day 24 | 20.30 | 6.21 |

The patient’s renal function was normal.

SCr: Serum creatinine; BUN: blood urea nitrogen.

Table S3: Coagulation function.

| Date | PT s | APTT s | D-Dimer mg/L | INR | FIB g/L | FDP mg/L |
| --- | --- | --- | --- | --- | --- | --- |
| Day 24 | 17.2 | Prolonged, no specific value | 0.10 | 1.67 | 1.36 | 0.7 |

The coagulation function was associated with the infected condition and the result did not point out a disseminated intravascular coagulation.

PT: prothrombin time; APTT: activated partial thromboplastin time; INR: international normalized ratio; FIB: fibrinogen; FDP: fibrinogen degradation products.
